# New streams and springs after the 2014 Mw6.0 South Napa earthquake

**DOI:** 10.1038/ncomms8597

**Published:** 2015-07-09

**Authors:** Chi-Yuen Wang, Michael Manga

**Affiliations:** 1 Department of Earth and Planetary Science, University of California, Berkeley, California 94720, USA

## Abstract

Many streams and springs, which were dry or nearly dry before the 2014 Mw6.0 South Napa earthquake, started to flow after the earthquake. A United States Geological Survey stream gauge also registered a coseismic increase in discharge. Public interest was heightened by a state of extreme drought in California. Since the new flows were not contaminated by pre-existing surface water, their composition allowed unambiguous identification of their origin. Following the earthquake we repeatedly surveyed the new flows, collecting data to test hypotheses about their origin. We show that the new flows originated from groundwater in nearby mountains released by the earthquake. The estimated total amount of new water is ∼10^6^ m^3^, about 1/40 of the annual water use in the Napa–Sonoma area. Our model also makes a testable prediction of a post-seismic decrease of seismic velocity in the shallow crust of the affected region.

## Abstract (web summary)

Following the Mw6 South Napa earthquake in California, previously dry streams and springs began to flow. Here, the authors present data from repeated stream surveys and laboratory measurements and suggest that the new flows originated from groundwater in the mountains and were released by the earthquake.

## Graphical abstract

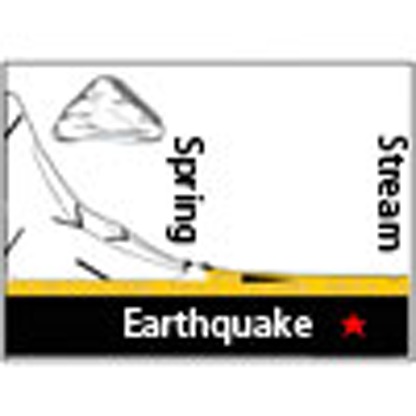

It has been known for millennia that earthquakes can induce a variety of hydrological responses. Pliny^1^ wrote about new flows after earthquakes almost 2,000 years ago, and many hypotheses have since been proposed to explain their origin^2^,^3^,^4^,^5^,^6^,^7^,^8^,^9^,^10^,^11^,^12^,^13^,^14^. Such streamflow increases are more than curiosities because understanding their origin can provide insight into the interactions between hydrogeologic and tectonic processes at spatial and temporal scales that are otherwise difficult to study. In addition, such understanding may have implications for underground waste repositories^15^, hydrocarbon production^16^, water supplies^1^,^17^ and subsurface transport of heat and solutes^18^.

The Mw6.0 South Napa earthquake of 24 August 2014, the largest earthquake to hit the San Francisco Bay area since the 1989 Mw6.9 Loma Prieta earthquake, occurred at a depth of 11 km on a steeply dipping, NNW striking branch of the West Napa fault zone^19^ (Fig. 1). At least nine tributary streams of the Napa River and Sonoma Creek and several springs in the Napa–Sonoma area, which were dry or had little flow before the earthquake, started to flow after the earthquake. In addition, a United States Geological Survey (USGS) stream gauge on Sonoma Creek documented a coseismic increase of discharge, showing that increased groundwater flow occurred across the basin after the earthquake. Since water in the new streams hardly mixed with pre-existing surface waters, its composition reflects the newly discharged water and provides a unique opportunity to identify its origin.

Following the earthquake we repeatedly surveyed the new flows, collected water samples and carried out laboratory measurements. Here we report the results of the survey, the origin of the new water and the amount of water released by the earthquake.

## Results

### Hydrogeologic setting of the studied area

**Figure 1:**
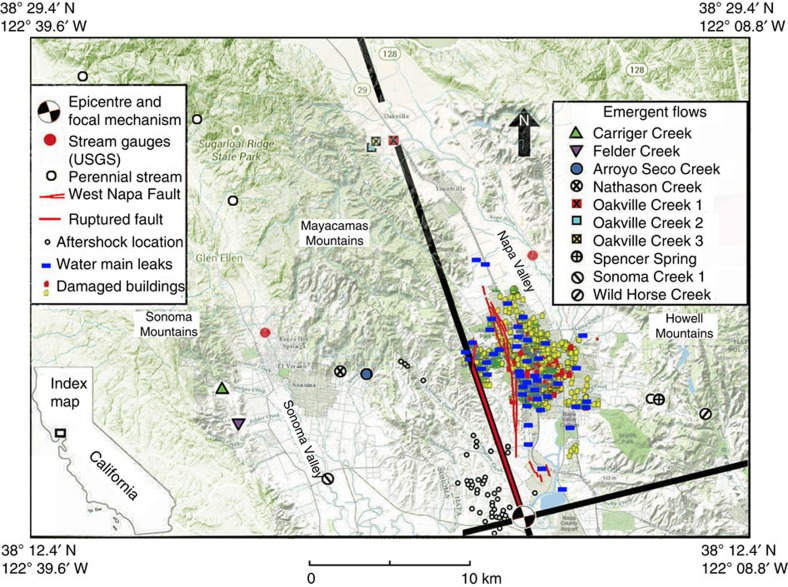
Map showing the study area. Included are locations of new streams, a pre-existing spring (Spencer Spring) with increased flow after the 2014 Mw6.0 South Napa earthquake, monitored sites of perennial streams, epicentre and focal mechanism of the earthquake, epicentres of aftershocks, USGS stream gauges, broken water mains and damaged buildings. Among the two USGS gauges, only the one on Sonoma Creek is studied here; the other gauge on Napa River was too close to two broken water mains after the earthquake to provide reliable data and is thus excluded from the study. Two additional sites on Sonoma Creek are denoted, respectively, as Sonoma Creek 1 (this figure) and Sonoma Creek 2 (Supplementary Tables 2 and 3 and Supplementary Note 1). Red lines show the West Napa fault zone; thick red line shows the ruptured fault^19^. The focal mechanism of the earthquake, shown by a ‘beach ball' symbol, divides the surrounding region into quadrants of static compression and dilatation, bounded approximately by the thick black lines marked on the map. Areas extending from the white sections of the ‘beach ball' are in static dilatation; areas extending from the black sections are in static compression. Notice that Spencer Spring is located in a dilatational quadrant and the Oakville Creeks are all located near the boundary between dilatational and compressional regimes. Index map at lower left corner shows the location of the studied area.

The studied streams and spring are all located in the Napa and Sonoma Valleys (Fig. 1; see also Supplementary Note 1 for coordinates and elevations of sampling sites), which are structural troughs between the NNW-trending Coast Ranges north of San Francisco Bay. The valleys are filled with Quaternary alluvial deposits that overlie Plio-Pleistocene sedimentary rocks, which in turn overlie Miocene to Pliocene Sonoma volcanics of uncertain thickness^20^,^21^. The surrounding mountains consist mostly of the Sonoma volcanics, with soil cover rarely exceeding a metre in thickness^22^.

Napa River and Sonoma Creek, the two main streams draining the respective basins, are fed by tributaries from the surrounding mountains. The average annual precipitation over the past 50 years has been about 72 cm. Most precipitation occurs between late autumn and early spring. The South Napa earthquake occurred near the end of a long dry season when many tributary streams and springs were dry.

**Figure 2:**
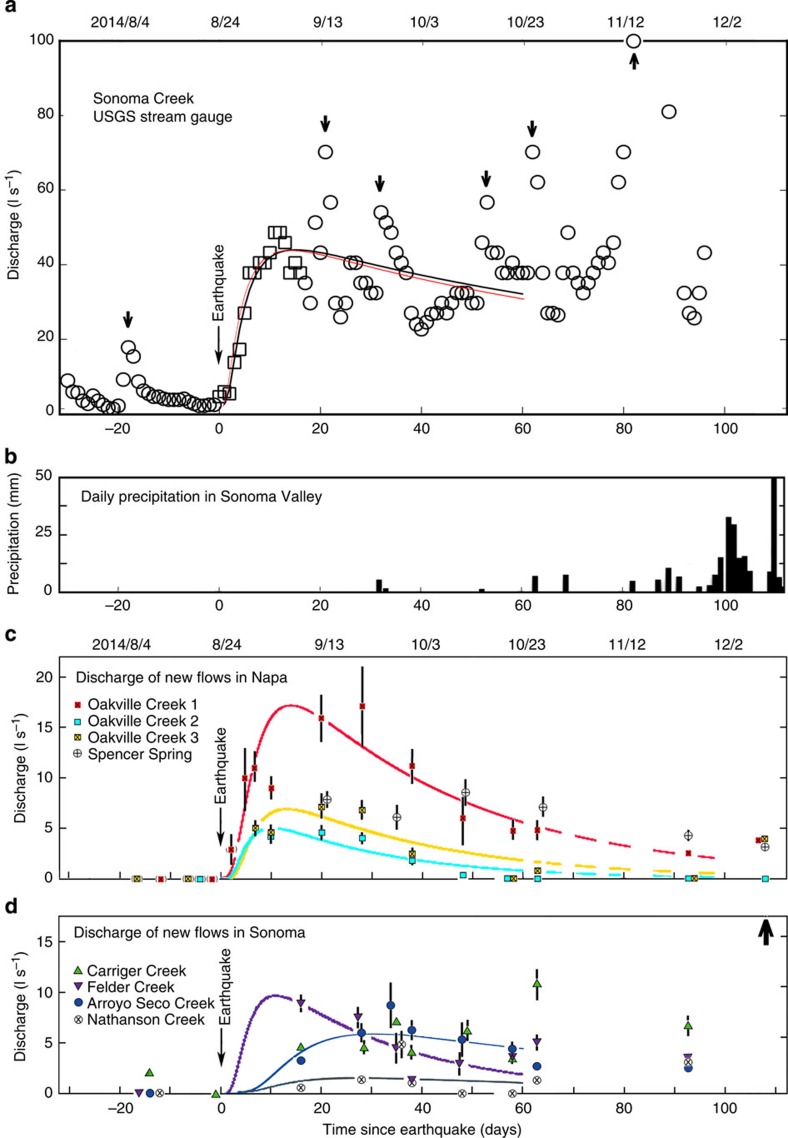
Changes in discharge after the South Napa earthquake. (**a**) Daily averaged discharge of Sonoma Creek documented by a USGS gauge at Agua Caliente before and after the South Napa earthquake. Measurement errors are similar to the size of symbols used. Short duration increases of discharge, indicated by thick arrows, were due to precipitation both inside and outside of the studied area, and do not necessarily correspond to the precipitation in the valley (**b**). Thin arrow shows the time of the earthquake. Simulations of the flow are based on the model discussed in the text and Supplementary Note 2. Two simulations were made; the first simulation (black line) is based on data for the first 17 days after the earthquake (open squares) to exclude the first incursion of precipitation and the second simulation (red line) is based on all data before significant precipitation in the valley (∼60 days after the earthquake). The similarity between the two results suggests robustness of model. (**b**) Daily precipitation in Sonoma Valley; precipitation in Napa Valley (not shown) was similar. Little precipitation occurred for 60 days after the earthquake, but significant precipitation started afterwards. (**c**) Discharges as a function of time in new streams and Spencer Spring in Napa County. Discharges in different streams and spring are shown by different coloured symbols; measurement errors are shown as error bars except where the error bars are smaller than the symbols. Symbols in brackets show conditions reported by local residents and one discharge data converted from early depth measurements, with depth-to-discharge conversion calibrated during subsequent surveys. Coloured curves show simulated stream discharges based on 60 days of data after the earthquake, using the same model as used in **a**; dashed curves show extrapolations from the simulated discharges. Measurement at Spencer Spring started 21 days after the earthquake; this flow is not simulated. (**d**) Discharges and simulated discharges as a function of time in new streams in Sonoma County. Upward arrow indicates that measured discharges were off scale.

Most new flows occurred near the base of mountains (Fig. 1) at elevations <200 m (Supplementary Note 1) and appeared only along a limited stretch of the channel. The sources of the flow, however, are mostly on private properties and inaccessible to this study. The only exception is Spencer Spring (Fig. 1), where water emerges directly from the ground at an elevation of ∼150 m and its discharge increased several fold after the earthquake. Two sources of information allowed us to ascertain that the selected streams and springs were either dry or had little flow before the earthquake. First is a monthly record of stream discharge in Sonoma, archived by the Sonoma Ecology Center. Comparing the records before and after the earthquake shows about 40% of the monitored dry streams before the earthquake started to flow after the earthquake. Second are reports by local residents close to the streams and springs. An example is an email from a resident in Oakville on 26 August: ‘… We have water flowing in our seasonal creek. Neighbour said he's never seen this in 70 years. I agree. Have been on our property for 24 years. Water is 2″ to 3″ deep and flowing…' Water in the stream was 8″ deep when we visited on 28 August, suggesting a gradual increase in discharge, similar to that documented at the Sonoma Creek by the USGS stream gauge (Fig. 2a).

### Discharge and temperature of new flows

Discharge of new flows was measured with standard methods (Supplementary Table 1). Figure 2a shows the daily average of stream discharge documented by the USGS stream gauge on Sonoma Creek. At higher sampling rate, stream discharge showed increased diurnal fluctuation after the South Napa earthquake (Supplementary Fig. 1), similar to that documented after the 2010 Maule (Chile) earthquake^12^, which could be caused by enhanced transpiration as plants have access to more water.

The post-seismic discharge peaked within 10–30 days after the earthquake (Fig. 2a,c,d) and decreased thereafter; some (Nathanson Creek and Oakville Creeks 2 and 3, for locations see Fig. 1) vanished ∼60 days after the earthquake.

Water temperature in the streams ranges from 13 to 21 °C (Supplementary Table 2); daytime air temperature in the valleys varies between 14 and 32 °C. Water temperature of Spencer Spring is nearly constant at 30–31 °C. Because the average surface temperature is ∼15 °C and the regional average geothermal gradient is 46 °C km^−1^ (ref. 21), at least part of the new water came from depths greater than ∼300 m beneath the surface assuming the average temperature gradient at the spring site.

### Stable isotope constraints

**Figure 3:**
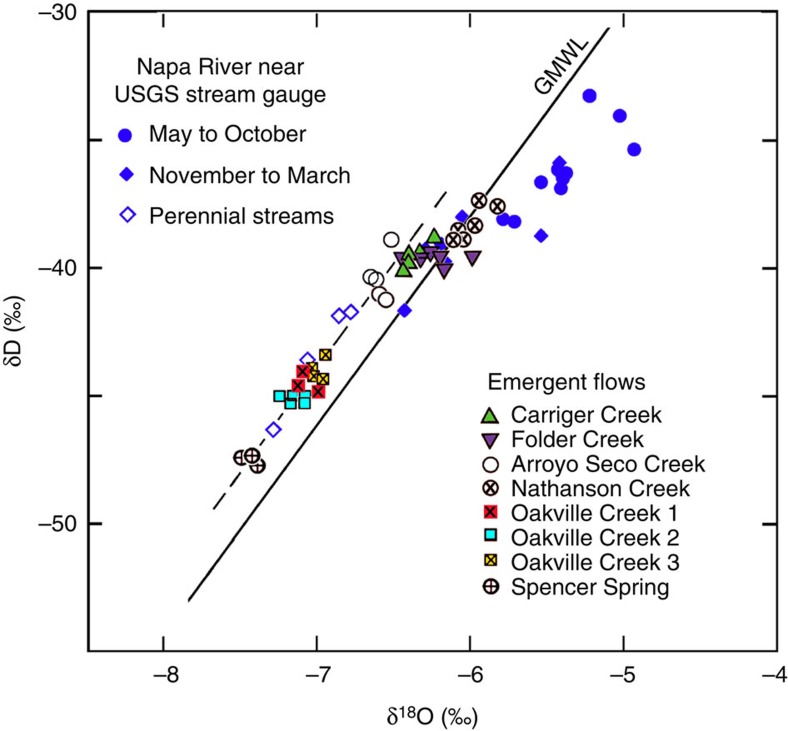
Stable isotope data for the studied streams and spring. Shown are measurements of δD versus δ^18^O for the new streams and Spencer Spring, the Napa River from 1984 to 1987, and three major perennial streams in foothills. Measurement errors are smaller than the size of symbols used. Solid line shows the GMWL. Data from this study define a local meteoric water line parallel to, but shifted slightly to the left of the GMWL. Notice that the isotopic compositions of each flow, sampled at different times (Supplementary Table 3), cluster together, while the isotopic compositions of different flows span a broad range along the local meteoric water line. During rainy seasons (normally November to March) the isotopic composition of the Napa River falls mostly close to the GMWL; during dry seasons, the Napa River composition becomes significantly heavier and falls to the right of the line due to evaporation and recharge by evaporated surface water.

**Figure 4:**
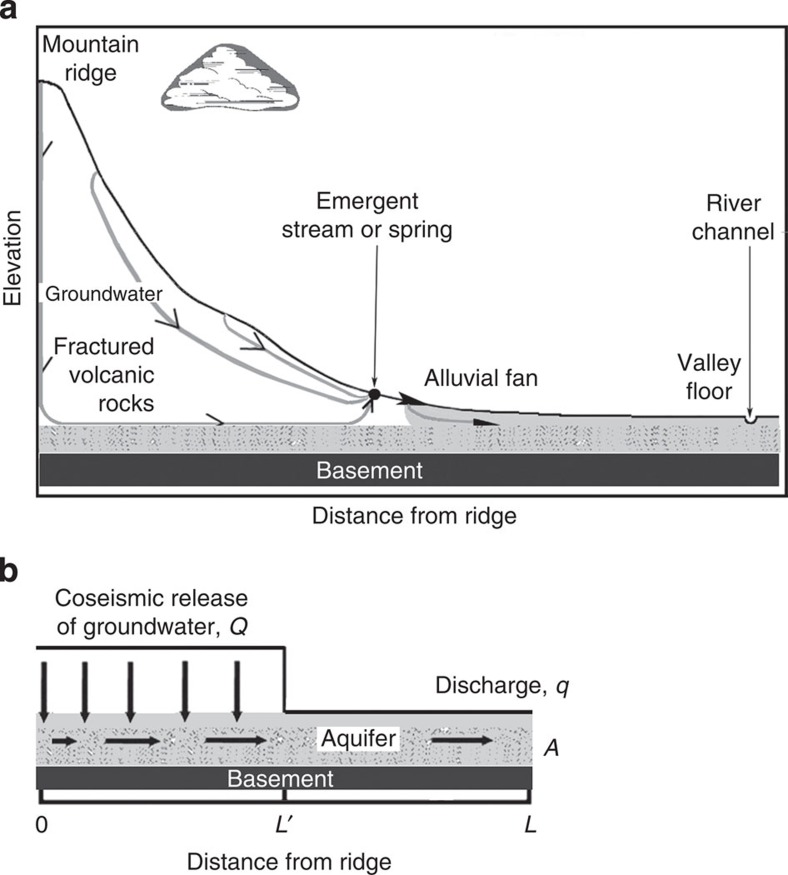
Conceptual model for the origin of new flows. (**a**) Cartoon showing the conceptual model of groundwater released by earthquake from nearby mountains to recharge an underlying aquifer. Precipitation that recharges groundwater in the mountain becomes isotopically lighter at higher elevation. (**b**) Diagram showing idealized conceptual model, adapted from **a**, for analytical simulation (Supplementary Note 2). *L* and *A* are, respectively, the length and cross-sectional area of the aquifer, *L′* the length of the recharged section of the aquifer, *Q* and *q*, respectively, the coseismic release of groundwater from mountain and the discharge from aquifer. Note that *L* and *L′* may be highly variable among streams; thus the diagram is intentionally drawn at a different scale from **a**.

The stable isotopes of hydrogen and oxygen of the new waters (Supplementary Table 3) define a linear relation on a δD versus δ^18^O plot (Fig. 3), parallel to, but slightly shifted to the left of, the global meteoric water line (GMWL)^23^. The slight shift from GMWL may reflect differences in humidity and temperature that affect secondary evaporation as rain falls from clouds^24^. The isotopic compositions of each flow, sampled at different times (Supplementary Table 3), cluster closely together (Fig. 3), suggesting that each flow came from a distinct source of constant composition. Different flows, on the other hand, span a broad range of isotopic composition (Fig. 3), suggesting different sources recharged by meteoric water at different elevations (Fig. 4a). Also plotted are the isotopic compositions of the Napa River determined at various times of year from 1984 to 1987 (ref. 25). From November to March, normally the rainy season, the isotopic composition of Napa River falls mostly close to the GMWL; during dry seasons, on the other hand, it becomes significantly heavier and falls to the right of the GMWL. The latter may reflect the evaporation of river water and recharge from shallow groundwater or reservoirs in the valley during dry seasons^21^.

## Discussion

Changes in crustal static strain^3^ have been proposed to explain the origin of the increased flows. To test this hypothesis, we note that the focal mechanism of the South Napa earthquake delineates the affected area into quadrants of static compression and dilatation (Fig. 1). If the new flows were caused by static poroelastic strain, no new flows would have appeared in the dilatational quadrants. However, Spencer Spring is located in a dilatational quadrant, and the Oakville Creeks are all located near the boundary between dilatational and compressional regimes (Fig. 1) where the volumetric strain is nearly zero. Thus the prediction of the hypothesis of static poroelastic strain is not consistent with observations.

For the dynamic stress hypothesis, four sources of water have been proposed that are consistent with the regional geology: consolidation and liquefaction of loose sediments in the valley^8^,^9^, water shaken out of the unsaturated zone (soils)^12^,^13^, rupturing of geothermal reservoirs or opening of deep fractures^10^,^11^ and groundwater in mountains released by seismic shaking^4^,^5^,^6^,^7^. Significant liquefaction was not observed following the South Napa earthquake. In addition, most of the new flows are isotopically much lighter than that of Napa River during dry seasons (Fig. 3) thus chemically distinct from shallow groundwater that recharges the river, refuting the hypothesis that consolidation or liquefaction of sediments could be the source of the new water. Soil water in Napa is also isotopically heavy^26^ and is unlikely a source of the new flows.

Fluids of inferred hydrothermal origin in Napa and Sonoma Valleys appear to remain close to the meteoric water line^27^. We measured the concentration of selected cations in collected water samples; among these, B and Li are the most useful for classifying waters and identifying their origins^27^. We list in Supplementary Table 4 the mean concentrations of B and Li of the studied flows and compare these with those in hydrothermal fluids and groundwater in Sonoma and Napa^27^. In general the concentrations of B and Li in the new streams are much more similar to the regional groundwater than to hydrothermal fluids. Even Spencer Spring does not show a strong geochemical signature of hydrothermal fluids although its relatively high and nearly constant temperature suggests a significant hydrothermal contribution.

Thus we are left with one hypothetical source for the new streams, that is, groundwater in the nearby mountains. The light isotopic composition of the new streams (Fig. 3) is consistent with the hypothesis that they originated from meteoric water precipitated at high elevations and stored as groundwater until being released through enhanced permeability caused by the earthquake^5^ (Fig. 4). The spread of δ^18^O in the new waters is about 1.5‰ (Supplementary Table 3). Assuming a global lapse rate of 2.1‰ km^−1^ (ref. 28), this spread corresponds to a difference in elevation of ∼700 m, which may be compared with the difference in elevation between the valley floor (near sea level) and some mountains in the studied area, such as Cobb Mountain at 1,440 m above sea level, and Mount Saint Helena and Hood Mountain over 762 m above sea level. It is also significant that the isotopic composition of some perennial streams in foothills (see Fig. 1 for sampling sites) falls along the same local meteoric water line defined by the new flows (Fig. 3). Since these streams are recharged by baseflow in mountains during dry seasons, the new flows are also likely to have originated from groundwater in mountains.

If the new flows originate from groundwater in the mountains, we can estimate the total amount of water released by the earthquake. Results of simulation of groundwater flow based on the conceptual model^5^ (Fig. 4a,b) that earthquakes may release groundwater in mountains by enhancing the vertical permeability are consistent with observed changes in discharge in several settings^5^,^12^,^14^. While the model is greatly idealized, it has been shown to be useful for estimating some first-order parameters of the process. From fitting the model to the coseismic discharge at the Sonoma Creek USGS stream gauge (Fig. 2a, see Supplementary Note 2 for details), we estimate the amount of new water to be 0.5 × 10^6^ m^3^ (Supplementary Table 5). Since this gauge is located upstream of all the new streams in Sonoma County, its discharge does not include the contributions from the new streams we monitored. By fitting the model to the discharge in all new flows (Fig. 2c,d), we estimate an additional amount of new water of 0.3 × 10^6^ m^3^ (Supplementary Table 5). Thus the total amount of new water discharged after the earthquake is of order 10^6^ m^3^. Part of the earthquake-released water may have infiltrated to rewet streambeds after a dry period, and the actual amount of water released may be greater than that estimated from the discharge data. The new discharges are an order of magnitude smaller than similar discharges after the 1989 M6.9 Loma Prieta earthquake in central California^29^, and about one 40th of the current annual water demand in the Napa–Sonoma region^21^,^30^, hardly a significant ‘water bonanza'^31^ to help the drought in the area.

Since drainage of groundwater from pores and fractures would make the shallow crust more compressible, our model predicts a post-seismic decrease of seismic velocity in the shallow crust of the region, which is consistent with reports from Japan following major earthquakes^32^ and preliminary results from the Napa area following the earthquake^33^.

## Additional information

**How to cite this article:** Wang, C.-Y. & Manga, M. New streams and springs after the 2014 Mw6.0 South Napa earthquake. *Nat. Commun.* 6:7597 doi: 10.1038/ncomms8597 (2015).

## Supplementary Material

Supplementary Information Supplementary Figure 1, Supplementary Tables 1-5, Supplementary Notes 1-2 and Supplementary References
