# Supplementary material for: New streams and springs after the 2014 Mw6.0 South Napa earthquake
Source: Nat Commun. 2015 Jul 9;6:7597. doi: 10.1038/ncomms8597 (PMC4510671; doi:10.1038/ncomms8597)
Supplement: Supplementary Information — Supplementary Figure 1, Supplementary Tables 1-5, Supplementary Notes 1-2 and Supplementary References [file ncomms8597-s1.pdf]

Supplementary Information:

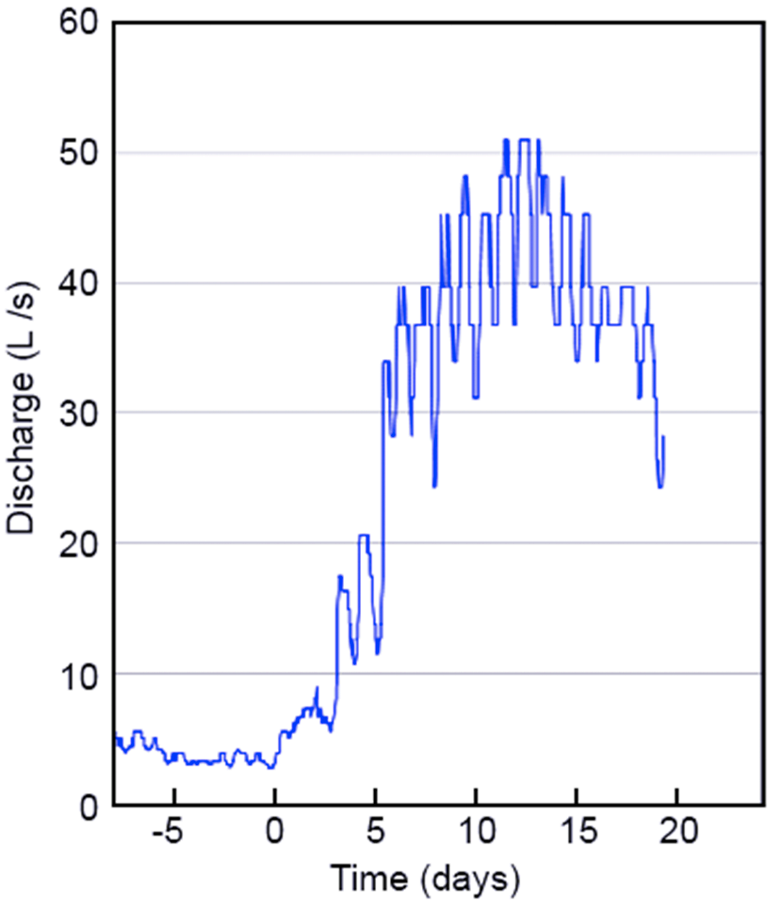

Supplementary Figure 1. Approved stream discharge of Sonoma Creek in L/s at the USGS stream gauge 11458500 (N38°19'24", W122°29'36"; see also Fig. 1 for location) recorded at 15-minute intervals. Time axis shows number of days before (negative) and after (positive) the South Napa earthquake. Stream discharge showed increased diurnal fluctuations after the South Napa earthquake.

Supplementary Table 1. Discharge (L/s) in new streams and a spring; measurement errors are listed directly below the measured discharge. Dashes indicate no measurement. Velocity measurements at each site were made where the channel had the straightest thalweg and rectangular cross-section. Discharge of the stream is determined from the product of the cross-sectional area and the velocity of the flow. Most velocity measurements were made using a float. About 10 readings were made for each measurement at a given site, mostly along different paths of the cross section. A flow meter was used to calibrate the conversion of float velocity to the mean velocity, yielding a conversion factor of 0.65, nearly identical to published conversion factor for shallow streams[1]. Measurements marked with stars were made by the Sonoma Ecology Center using a flow meter (Marsh-McBirney, Model 2000, accuracy 2%). One datum marked with ^ was from ref 2. Data in parentheses were reported by local residents.

Date	Carriger Creek	Felder Creek	Arroyo Seco Creek	Nathanson Creek	Oakville Creek 1	Oakville Creek 2	Oakville Creek 3	Spencer Spring
07/15*	0.5	(pool)	(damp)	dry	(dry)	---	(dry)	---
08/07*	2.3	(dry)	(dry)	(dry)	(dry)	---	(dry)---	---
08/23	dry^	---	---	---	(dry)	---	(dry)	
08/26	---	---	---	---	(~3)<br>±1.5	---	---	---
08/28	---	---	---	---	10<br>±4	---	---	---
08/31	---	---	---	---	11<br>±1.6	---	5.0<br>±0.7	---
09/03	---	---	---	---	9.1<br>±1.3	4.4<br>±0.6	4.6<br>±0.7	---
09/09*	4.7<br>±0.5	8.7<br>±0.9	3.4<br>±0.3	0.6<br>±0.1	---	---	---	---
09/13	---	---	---	---	16<br>±2.4	4.6<br>±0.7	7.2<br>±1.1	---
09/14	---	---	---	---	---	---	---	7.8<br>±1.0
09/21	4.7<br>±0.7	7.5<br>± 1.1	6.1<br>± 0.9	1.4<br>± 0.2	17.1<br>± 4.0	4.1<br>± 0.6	6.9<br>± 1.0	---
09/28	6.5<br>±0.3	4.6<br>± 1.6	8.7<br>± 2.2	4.9<br>± 1.5	---	---	---	6.1<br>± 1.0
10/01	4.2<br>±0.6	1.6<br>± 0.2	6.4<br>± 1.0	1.2<br>± 0.2	11.3<br>± 1.7	1.8<br>± 0.3	2.5<br>± 0.4	---
10/11	6.1<br>±0.7	3.1<br>± 1.3	5.5<br>± 2.1	0.1<br>±0.03	6.1<br>±2.5	0.4<br>± 0.03	---	8.6<br>± 1.4
10/21	3.6<br>±0.5	3.7<br>± 0.6	4.5<br>± 0.7	(pool)	4.8<br>± 1.0	(dry)	(dry)	---

10/26	10.9<br>±1.6	5.1<br>± 0.8	2.7<br>± 0.4	1.3<br>± 0.2	4.8<br>± 1.0	(dry)	0.8<br>± 0.1	---
10/27	---	---	---	---	---	---	---	7.1<br>±1.0
11/24	6.7<br>±1.0	3.6<br>± 0.5	2.7<br>± 0.4	3.2<br>± 0.5	2.6<br>± 0.4	(dry)	(dry)	4.3<br>± 0.6
12/10	31.0<br>±6.0	20.5<br>± 3.0	20.7<br>± 2.1	---	3.9<br>± 0.5	(dry)	4.0<br>± 0.6	3.4<br>± 0.5

Supplementary Table 2. Temperature (ºC) in studied streams and spring; dashes indicate no measurement. Temperature of water was measured with a thermistor with an accuracy of 0.1 ºC. The thermistor was immersed in flowing water until thermal equilibration is established. Air temperature was not documented because thermal equilibrium between the thermistor and air was difficulty to establish.

Date	Carriger Creek	Felder Creek	Arroyo Seco Creek	Nathanson Creek	Oakville Creek 1	Oakville Creek 2	Oakville Creek 3	Spencer Spring
08/28	---	---	---	---	21.3	---	---	---
08/31	---	---	---	---	21.1	---	20.9	---
09/03	---	---	---	---	19.7	19.3	19.2	---
09/09*	15.6	17.2	16.7	17.2	---	---	---	---
09/13	19.0	17.7	17.3	20.4	17.6	17.6	18.6	---
09/14	---	---	---	---	---	---	---	30.2
09/21	17.5	17.0	17.6	18.5	18.2	18.6	18.0	---
09/28	17.4	15.8	17.1	18.5	---	---	---	30.4
10/01	17.8	16.3	16.8	18.5	17.0	18.2	17.4	---
10/11	17.5	15.4	16.4	16.9	16.0	16.6	---	30.8
10/21	15.3	13.5	15.6	15.2	17.1	---	---	---
10/26	14.6	13.4	15.4	14.8	16.8	---	16.2	---
10/27	---	---	---	---	---	---	---	30.9
11/24	12.4	10.3	12.7	10.6	11.9	---	---	31.0
12/10	13.1	13.9	13.9	14.0	15.2	---	13.7	31.0

Perennial streams (See Fig. 1 and Supplementary Note 1 for sampling site locations):

	Sonoma Creek 2	Calabazas Creek	Santa Rosa Creek	Spencer Creek
Date	09/03/2014	09/03/2014	09/03/2014	09/14/2014
T(ºC)	15.8	16.7	15.7	23.2

Supplementary Table 3. Laboratory determination of the oxygen and hydrogen isotopic compositions of water samples collected from emergent flows on different dates. For each determination, $\delta^{18}O$ is listed on the upper row and δD on the lower row; dashes indicate no determination. Analysis was performed at UC Berkeley's Center for Isotope Biogeochemistry, using ThermoQuest Finnigan Delta plus XL isotope ratio mass spectrometer along with Dual Inlet, H/Device (HD) and GasBanch (GB). The O-isotope composition is determined by H_2O-CO_2 equilibrium GB method and the analytical precision is better than 0.05‰. The H-isotope composition is determined by Chrome HD method and the analytical precision is better than 0.6‰.

Date	Carriger Creek	Felder Creek	Arroyo Seco Creek	Nathanson Creek	Oakville Creek 1	Oakville Creek 2	Oakville Creek 3	Spencer Spring
08/28	---	---	---	---	-7.14<br>-44.9	---	---	---
08/31	---	---	---	---	-7.14<br>-45.0	-7.21<br>-45.3	-7.01<br>-44.0	---
09/03	---	---	---	---	-7.15<br>-45.1	-7.17<br>-45.3	-6.96<br>-44.4	---
09/09	-6.26<br>-39.4	-6.23<br>-38.8	-6.51<br>-38.9	-5.95<br>-37.3	---	---	---	---
09/13	-6.32<br>-39.6	-6.33<br>-39.5	-6.61<br>-40.5	-5.82<br>-37.6	-7.24<br>-45.0	-7.03<br>-43.9	---	---
09/14	---	---	---	---	---	---	---	-7.48<br>-47.4
09/21	-6.44<br>-39.6	-6.32<br>-39.6	-6.64<br>-40.4	-6.07<br>-38.5	-7.14<br>-44.9	-7.15<br>-45.0	-7.01<br>-44.2	---
09/28	-6.37<br>-39.9	-6.17<br>-39.6	-6.52<br>-41.3	-6.08<br>-39.0	---	---	---	-7.37<br>-47.8
10/01	-6.37<br>-39.6	-5.89<br>-39.7	-6.56<br>-41.1	-6.02<br>-39.0	-7.06<br>-44.1	-7.06<br>-45.4	-6.92<br>-43.5	---
10/11	-6.41<br>-40.2	-6.13<br>-40.1	-6.56<br>-41.1	-5.94<br>-38.4	-6.98<br>-44.9	-7.05<br>-45.2	-7.08<br>-45.2	-7.42<br>-47.4

Perennial streams (See Fig. 1 and Section 1 for locations of sampling sites):

	$\delta^{18}O$	δD
Sonoma Creek 2, 09/03/14	-6.85	-41.9
Calabazas Creek, 9/03/14	-7.06	-43.6
Santa Rosa Creek, 9/03/14	-6.78	-41.7
Spencer Creek, 9/14/14	-7.29	-46.4

Supplementary Table 4: Laboratory determination of composition of water samples collected from emergent flows on different dates, together with the mean concentrations of Li and B in hydrothermal fluids[3] in Sonoma and Napa Valleys for comparison. Measurements were made on filtered water samples by ICP-MS at the Lawrence Berkeley National Lab. The differences between repeated measurements on different aliquots of water for Li average about 5%, and 15% for B.

Sample	Date	Li (ppb)	B (ppb)
Oakville 1	10/1/14	27.1	431
	10/11/14	27.8	447
Oakville 2	10/1/14	32.6	587
	10/11/14	38.1	467
Oakville 3	10/1/14	9.7	537
	10/11/14	27.5	574
Felder	10/1/14	27.7	92
	10/11/14	28.4	96
Arroyo Seco	10/1/14	8.3	43
	10/11/14	9.1	48
Carriger	10/1/14	3.6	18
	10/11/14	3.7	19
Nathanson	10/1/14	1.8	18
	10/11/14	1.8	18
Spencer Spring	10/11/14	79.4	739
*Hydrothermal fluid		1633	10723
*Groundwater		15.5	211

* Concentrations of Li and B in hydrothermal fluid and groundwater are from ref 3.

Supplementary Table 5. Model parameters (upper row) and their uncertainties (lower row) determined from model simulation of stream discharge (see Supplementary Note 2 and text for details of the simulation).

Stream	Q (m^3)	L'/L	D/L^2 (day^{-1})
Sonoma Creek	5.3 $x10^5$	0.70	0.0029
at USGS gauge	+/- 0.57 $x10^5$	+/- 0.00	+/- 0.0002
Oakville 1	0.87 $x10^5$	0.56	0.0075
	+/- 0.38 $x10^5$	+/- 0.22	+/- 0.005
Oakville 2	0.23$x10^5$	0.32	0.0099
	+/- 0.15 $x10^5$	+/- 1.5	+/- 0.010
Oakville 3	0.24$x10^5$	0.45	0.013
	+/- 0.09 $x10^5$	+/- 0.32	+/- 0.007
Carriger	0.60$x10^5$	0.27	0.0049 day^{-1}
	+/- 0.28 $x10^5$	+/- 0.54	+/- 0.002
Felder	0.35$x10^5$	0.1	0.014
	+/-0.05 $x10^5$	(set)	+/- 0.005
Arroyo Seco	0.61 $x10^5$	0.1	0.0054
	+/- 0.07 $x10^5$	(set)	+/- 0.0005
Nathanson	0.14 $x10^5$	0.1	0.0059
	+/-0.12 $x10^5$	(set)	+/- 0.005

Supplementary Note 1. Coordinates of field sites

Site name	Latitude	Longitude	elevation (m)
Sonoma Valley:			
Carriger Creek	N38°17.409’,	W122°31.352’	118[a]
Felder Creek	N38°16.140’,	W122°30.191’	38[a]
Arroyo Seco Creeek	N38°18.001’,	W122°25.485’	80[a]
Nathanson Creek	N38°18.051’,	W122°26.286’	67[a]
Napa Valley:			
Oakville Creek 1	N38° 25.948’	W122° 24.161’	53[b]
Oakville Creek 2	N38° 25.724’	W122° 25.000’	77[b]
Oakville Creek 3	N38° 25.930’	W122° 24.943’	70[b]
Spencer Spring	N38° 17.040’	W122° 12.890’	152[c]
Perennial tributary streams:			
Santa Rosa Creek	N38° 28.024’	W122° 37.306’	137[b]
Sonoma Creek 2	N38° 26.525’	W122° 32.416’	197[b]
Calabazas Creek	N38° 23.661’	W122° 30.963’	133[b]
Spencer Creek	N38° 17.092’	W122° 13.108’	136[d]

a. Elevation from tabulated data by the Sonoma Ecology Center
b. Elevation read from USGS topographic maps
c. Elevation read from google (by Heiko Woith and Tom Flesher)
d. Elevation estimated using GPS (by David Pert)

Supplementary Note 2. Model simulation and stream discharge

We simplify the conceptual model in Fig. 4a to the configuration shown in Fig. 4b in order to apply an analytical solution[5] (see text for discussion) to assess the excess stream discharge in response to a coseismic release of groundwater from mountains, with the latter caused by a coseismic enhanced vertical permeability.

The solution for the excess stream discharge is[5]

$$q = \frac{2DQ}{LL'} \sum_{r=1}^{\infty} (-1)^{r-1} \sin\left[\frac{(2r-1)\pi L'}{2L}\right] \exp\left[-\frac{(2r-1)^2 \pi^2 D}{4L^2} t\right]$$

where Q is the coseismic release of groundwater, L the length of the aquifer, L' the length of the recharged section of the aquifer, t the time since the earthquake, $D = K/S_s$ is the hydraulic diffusivity of the aquifer, K the hydraulic conductivity and S_s the specific storage. In the solution of the groundwater flow equation these parameters appear in three free parameters: Q, D/L^2 and

L'/L. We determine the parameters by fitting the equation to the time history of the observed excess flows using the non-linear least-squares Marquardt-Levenberg algorithm. Some of these parameters may contain large uncertainties in view of the relatively small number of data available. If uncertainties are so large that any one parameter is poorly determined, we fix *L'/L* to 0.1. The parameters so determined are listed together with their standard errors in Supplementary Table 5.

Assuming $L \sim 2$ km (half width of Sonoma Valley at the USGS stream gauge) and using $D/L^2 = 0.0029$ day^{-1} for Sonoma Creek from model simulation, we obtain $D \sim 0.1$ m^2/s at the basin scale.

Supplementary References

1. Hiscock, K.M. and V.F. Bense (2014), *Hydrogeology*, Wiley.

2. Kovner, G., Napa quake jumpstarts stream flows, though probably only temporarily, The Press Democrat, September 4, (2014).

3. Forrest, M. J., J. T. Kulongoski, M. S. Edwards, C. D. Farrar, K. Belitz, and R. D. Norris, Hydrothermal contamination of public supply wells in Napa and Sonoma Valleys, California, Applied Geochemistry, 33(0), 25-40, doi:http://dx.doi.org/10.1016/j.apgeochem.2013.01.012, (2013).

4. Wang, C.-Y., C. H. Wang, and M. Manga, Coseismic release of water from mountains: Evidence from the 1999 (Mw= 7.5) Chi-Chi earthquake, Geology, 32, 769–772, (2004).
